# Local Control of Advanced Breast Cancer—Debate in Multidisciplinary Tumor Board

**DOI:** 10.3390/jcm14020510

**Published:** 2025-01-15

**Authors:** Iuliana Pantelimon, Andra Maria Stancu, Simona Coniac, Andreea-Iuliana Ionescu, Dimitrie-Ionuț Atasiei, Dragoș Eugen Georgescu, Laurenția Nicoleta Galeș

**Affiliations:** 1 Department of Medical Oncology, Clinical Hospital Dr. Ion Cantacuzino, 030167 Bucharest, Romania; iuliana.pantelimon@umfcd.ro (I.P.); andra-maria.stancu@rez.umfcd.ro (A.M.S.); simona.horlescu@drd.umfcd.ro (S.C.); 2 Discipline of Medical Oncology, “Carol Davila” University of Medicine and Pharmacy, 020021 Bucharest, Romania; laurentia.gales@umfcd.ro; 3 Department of Physiology, Craiova University of Medicine and Pharmacy, 200349 Craiova, Romania; 4 Department of Endocrinology, Faculty of Medicine, “Carol Davila” University of Medicine and Pharmacy, 020021 Bucharest, Romania; 5 Department of Radiotherapy, Coltea Clinical Hospital, 030167 Bucharest, Romania; andreea-iuliana.miron@drd.umfcd.ro; 6 Discipline of Oncological Radiotherapy and Medical Imaging, “Carol Davila” University of Medicine and Pharmacy, 020021 Bucharest, Romania; 7 Department of Surgery, Clinical Hospital Dr. Ion Cantacuzino, Bucharest, 030167 Bucharest, Romania; dragos-eugen.georgescu@umfcd.ro; 8 Discipline of Surgery, “Carol Davila” University of Medicine and Pharmacy, 020021 Bucharest, Romania; 9 Department of Medical Oncology, Bucharest Institute of Oncology “Prof. Dr. Al. Trestioreanu”, 022328 Bucharest, Romania

**Keywords:** advanced breast cancer, CDK 4/6 inhibitors, radiotherapy, multidisciplinary team, quality of life

## Abstract

**Background/Objectives**: In Romania, breast cancer is the second most common cancer, the third leading cause of cancer death, and the most prevalent cancer overall. De novo advanced-stage breast cancer often presents in clinical practice, and treatment decisions are best made in a multidisciplinary tumor board (MTD) involving surgeons, radiotherapists, and medical oncologists. Significant advances in systemic therapies, particularly in progression-free survival (PFS) and overall survival (OS), have surpassed traditional palliative mastectomy and radiotherapy for local control. Therefore, the purpose of this study is to emphasize the importance of the initial choice of treatment for patient prognosis. **Methods**: We expose two cases of patients with de novo severe, advanced-stage, hormone receptor (HR)-positive, human epidermal growth factor receptor 2 (HER2)-negative breast cancer and their management and outcome using cyclin-dependent kinase (CDK) 4/6 inhibitor and radiotherapy. An extensive review of the literature from the past five years was also conducted. **Results**: The role of palliative mastectomy is diminishing, as many patients are opting for novel therapies, including cyclin-dependent kinase (CDK) 4/6 inhibitors, which may improve quality of life. **Conclusions**: First-line therapy for locally advanced breast cancer has suffered changes due to the implementation of systemic targeted therapy. However, drug resistance—either de novo or acquired—remains a critical consideration. MTD discussions and informed patient decisions are essential to achieving a personalized, evidence-based treatment outcome.

## 1. Introduction

Breast cancer (BC) is a common condition that has a considerable impact on the female day-to-day life. Globally, BC ranks second in incidence when stratified only by women. The Asian continent is the leading region in terms of incidence, mortality, and prevalence, followed by Europe. Although the incidence is high, the global mortality ranks only 4. These results can be translated as an effective treatment plan against the disease [1]. However, according to Globocan, deaths due to BC in Romanian females aged 20–85+ are projected to rise from 3.92 to 4.18 thousand between 2020 and 2040, reflecting a 6.8% increase. Incidence is also expected to climb by 2%, from 11.8 to 12.1 thousand new cases. The age-standardized mortality rate (ASR) for 2020 was 28.9 per 100,000, placing Romania seventh in Europe [2]. In 2020, the International Agency for Research on Cancer reported breast cancer as the most prevalent, second most incident, and third most fatal cancer in Romania [3]. Consequently, BC is a key determinant of an active disease screening program in the healthcare system and efficient therapeutic options.

The existing body of research on BC suggests that, despite ongoing screening efforts, de novo advanced-stage breast cancer remains a frequent presentation [4,5]. The rationale lies in the misconception of the population regarding the disease. Even if the national and other screening programs are presented to the female inhabitants, the lack of time, old healing approaches, and the fear of the diagnosis are the main reasons for delayed therapy. Therefore, multidisciplinary tumor boards (MTDs) play a crucial role in determining appropriate initial treatment and ensuring comprehensive, sequential follow-up to optimize the benefits of local and systemic therapies [6,7]. In oncological therapy, there is still uncertainty regarding the targeted therapy. The efficacy of cyclin-dependent kinase (CDK) 4/6 inhibitors in combination with endocrine therapy (ET) for advanced or metastatic hormone receptor (HR)-positive, human epidermal growth factor receptor 2 (HER2)-negative breast cancer has sparked intense debate as an upfront local treatment [8,9,10,11]. Hence, a much-debated question is whether the use of palliative mastectomy or radiotherapy for bulky, bleeding tumors is beneficial in the actual context, particularly given the limited chemotherapy sensitivity of HR-positive and HER2-negative cancers. Supporters of surgery argue it offers immediate symptom relief and may improve survival, especially in places where radiotherapy is not an option. Critics, on the other hand, point out the physical and emotional tolls, like loss of sensation and body image issues. In contrast, CDK4/6 inhibitors paired with endocrine therapy provide a non-invasive way to manage disease progression and enhance quality of life. This contrast between surgical and systemic treatments highlights the need for personalized plans shaped by multidisciplinary tumor board (MTD) discussions to balance effectiveness, patient preferences, and overall well-being.

Considering the long-term management of advanced breast cancer, patient quality of life (QoL) and patient preferences are mandatory when establishing a treatment plan [12,13,14,15,16]. This review addresses key gaps in our understanding of local control strategies for advanced breast cancer. By delving into case studies and recent literature, we shed light on the critical interplay between systemic and local therapies discussed in multidisciplinary tumor board (MTD) meetings. Our goal is to show how these discussions shape personalized, evidence-based treatment plans that enhance both survival rates and quality of life. Through this analysis, we clarify the role of local control amidst the rise of advanced systemic therapies and suggest ways to effectively integrate these approaches into clinical practice. Therefore, the objective of this study is to present a real-world clinical experience, outlining the advantages and disadvantages of the actual treatment approaches in an MTD setting and patient-oriented fashion, in line with the National Comprehensive Cancer Network (NCCN) and European Oncology, Radiotherapy, and Breast Surgery guidelines.

## 2. Materials and Methods

The current study adopts a case study approach. Two female patients aged 64 and 69 years old with de novo advanced-stage breast cancer were referred to our clinic during 2021–2024. The patients’ performance status was evaluated according to the Eastern Cooperative Oncology Group (ECOG). Because of the severity of the disease, an MTD was established for an optimal treatment plan and follow-up. All patients were treated with endocrine therapy (ET) and CDK4/6 inhibitor without palliative mastectomy. Palbociclib was the inhibitor of choice, as approved by the European Medicines Agency (EMA) [17] and NIH-approved protocol of reimbursement, available on the Romanian National Society of Medical Oncology (SNOMR) website [18]. Treatment tolerance and adverse reactions were monitored and classified according to the Common Terminology Criteria for Adverse Events (CTCAE), version 5.0 [19]. This study was approved by the Ethics Committee of Cantacuzino Clinical Hospital, Bucharest, where it was conducted following their decision. Informed consent was obtained from all participating patients, who were thoroughly informed about the study’s purpose and assured that they and their data would not be exposed to any risks.

For the literature review, in February 2024, a thorough literature search was carried out using PubMed, Google Scholar, and Scopus. The focus was on studies from the past five years (February 2019–February 2024), using keywords such as “CDK4/6 inhibitor”, “de novo advanced-stage breast cancer”, “palliative mastectomy”, “breast cancer radiotherapy”, “tumor board in breast cancer”, “palliative surgery”, “local control in breast cancer”, and “quality of life in advanced-stage breast cancer.” Additional references were extracted from the articles retrieved during the search. Inclusion criteria were defined as studies published within the past five years, articles with abstracts and manuscripts written in English, and research that specifically addressed advanced-stage breast cancer. Exclusion criteria were studies published before February 2019, articles with abstracts and manuscripts not available or not in English, and research that did not specifically address advanced-stage breast cancer or related treatments. Since this was not a systematic review, the PRISMA flow chart was not applied.

## 3. Results

### 3.1. Case 1—G.S.

Patient G.S., female, 69 years old (y.o.), came to our Oncology Department in February 2023 for a bleeding tumoral mass localized in the left breast that progressed over several months.

**Figure 1:**
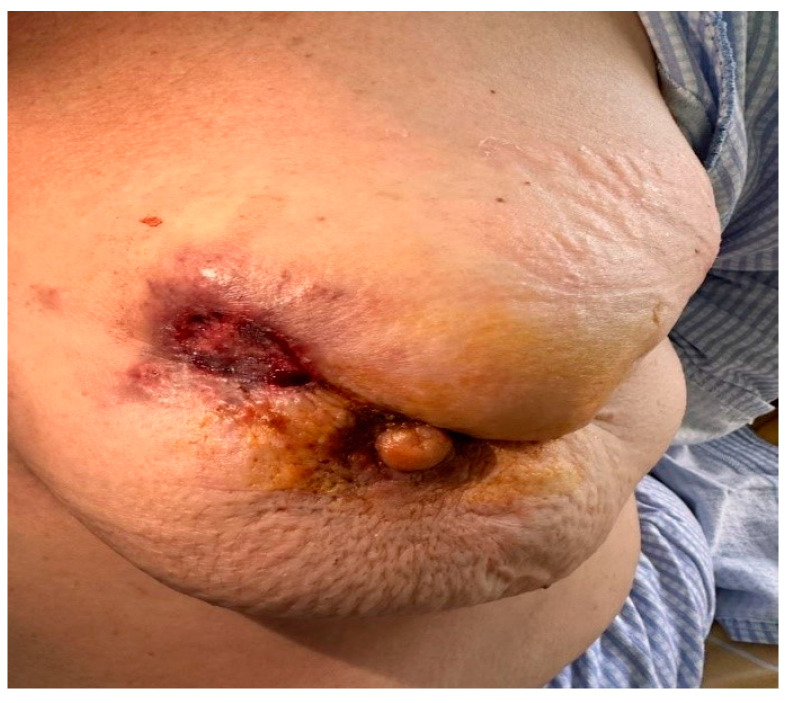
Clinical image of Case 1—G.S. The figure illustrates approximately a 10 cm ulcerated, with active bleeding, tumor in the central quadrant of the left breast; adjacently, skin invasion with retraction can be observed.

Clinical examination showed a 10 cm ulcerated, bleeding tumor in the central quadrant of the left breast, along with multiple 2 cm axillary lymph nodes. The patient uttered moderate pain in the left breast and non-specific bone pain. She was obese, with a body mass index (BMI) of 35, on anti-hypertensive treatment, and postmenopausal; additionally, she had previously undergone hysterectomy and bilateral oophorectomy for uterine fibroids. There was no family history of cancer, no allergies, and no occupational exposure to carcinogens (Figure 1).

**Figure 2:**
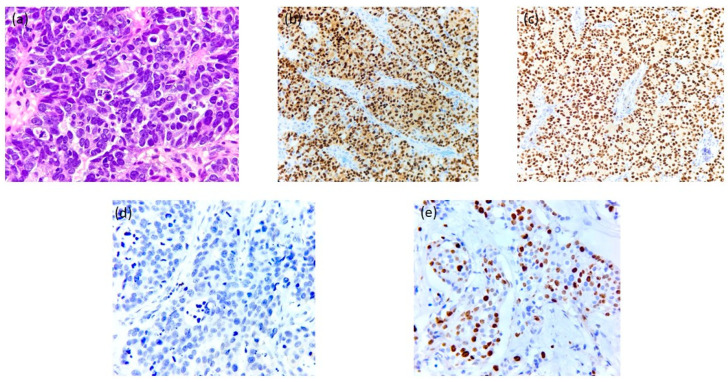
Immunohistochemistry of Case 1—G.S. (**a**) HE × 400: Invasive breast carcinoma NST (invasive ductal carcinoma NOS)-G3: sheets of large, pleomorphic tumor cells with frequent mitotic figures; (**b**) ER × 200: Estrogen receptor positive with strong nuclear expression in tumoral cells; (**c**) PR × 200. Progesterone receptor positive with strong nuclear expression in tumoral cells; (**d**) HER2 × 400: negative (no staining observed in tumor cells); (**e**) Ki67 × 200: Estimated Ki67 labeling index is ~70%.

The biopsy confirmed a pathological diagnosis of invasive ductal carcinoma, no special type (NST), poorly differentiated (G3), estrogen receptor (ER) positive, progesterone receptor (PgR) positive, and HER2-negative, immunohistochemistry (IHC) score = 0, with a Ki-67 proliferation index of 70% (Figure 2). Whole-breast ultrasound revealed a 7 cm tumor in the central quadrant of the left breast, extending to the upper quadrants, invading the skin with irregular borders and vascular signal. The tumor had deep infiltration into the pectoral muscle and adipose tissue. An additional 1.6 cm tumor with similar characteristics was found in the lower quadrants. The left axilla presented with multiple lymph nodes, the largest measuring 1.5 × 1 cm, displaying cortical asymmetry, while above the collarbone, another lymph node has been identified. A computed tomography (CT) scan of the thorax, abdomen, and pelvis showed carcinomatous mastitis in the left breast, axillary lymphadenopathy, and metastases to the lungs and bones. Whole-body bone scintigraphy confirmed multiple bone metastases, including lesions in the sternum, vertebral column, right rib cage, and right iliac wing. The final diagnosis was ER-positive metastatic breast cancer (mBC), cT4cN3c with pulmonary (M1Pul) and osseous metastases (M1Oss), stage IV.

The case was discussed with the MTD, and we concluded that local control would be better achieved through systemic therapy and radiotherapy (RT) rather than palliative surgery at this time. Therefore, the patient began ET with the aromatase inhibitor letrozole, 2.5 mg/day p.o., during RT. Palliative Radiotherapy for local control of the left breast and bone metastases was conducted. Volumetric-modulated arc therapy (VMAT), achieving a total dose of 30 Gy/10 fractions (3 Gy/fraction) on left breast target volume and a total dose of 30 Gy/10 fractions (3 Gy/fraction) on vertebral column bone metastasis. RT was well-tolerated with mild skin toxicity. Concurrent, systemic treatment with the CDK4/6 inhibitor Palbociclib was initiated.: 125 mg/day p.o. for 21 days in a 28-day cycle until the completion of RT. To prevent the progression of lytic bone lesions, delay skeletal-related events, and alleviate bone pain, we administered bisphosphonates in the form of zoledronic acid (15-min bolus, 4 mg/month). Additionally, the patient was treated by a dermatologist for local control; mild radiation dermatitis was treated with a moisturizer during and after RT. The tolerance for the CDK4/6 inhibitor was good. The CTCAE severity scale was occasionally grade 1, with neutropenia that did not require any treatment interruptions.

Based on the histological features of the tumor and the good tolerance of the treatment plan, our expectations included a significant improvement in local control of the BC through the combination of RT and systemic therapy. While RT aims to damage the cancer cells using precise radiation energy, the use of the aromatase inhibitor is anticipated to effectively target the estrogen-dependent nature of the cancer, whereas the CDK4/6 inhibitor, palbociclib, is expected to reduce the ulceration, bleeding, and skin retraction. Moreover, it should improve the other systemic involvements and slow the tumor progression while maintaining a good quality of life. Additionally, the administration of zoledronic acid should prevent skeletal-related events and manage bone pain associated with metastatic lesions.

**Figure 3:**
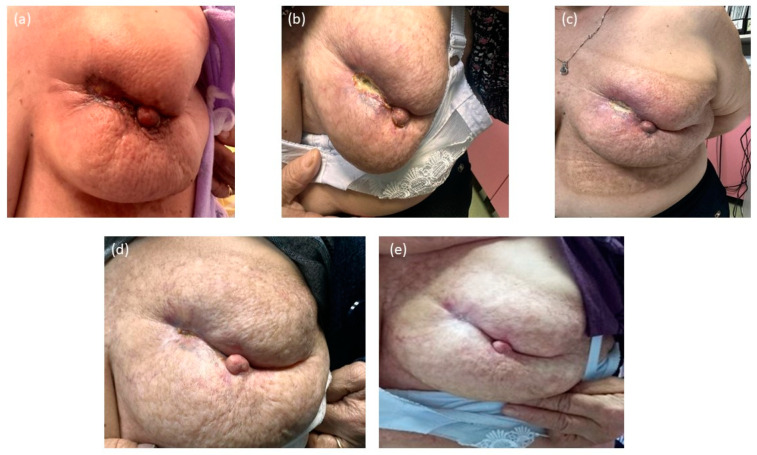
The clinical improvement of Case 1—G.S., after treatment completion, over a period of 12 months. (**a**) April 2023, two months post-therapy, the bleeding and ulceration show signs of healing, with no active bleeding and ulceration and reduced inflammation. (**b**) July 2023, a healing tissue begins to form, with significantly reduced inflammation and retracted skin. (**c**) August 2023, the healing process continued to develop, with the initial lesion becoming almost entirely covered by the healing tissue. (**d**) October 2023, the initial lesion is resolved, with only regions of healthy cicatricial tissue and skin remodeling due to the initial structural changes made by the tumor. (**e**) January 2024, the cicatricial tissue reduced its circumference, with healthy skin color and no signs of inflammation; post-radiation dermatitis skin can be observed across the region of RT.

From the clinical point of view, we achieved a great outcome with the treatment plan chosen in accordance with the MTD. Due to the patient’s good adherence to the treatment approach, her medical condition has significantly improved. During the follow-up period, the ulceration resolved in increments while the bleeding stopped. Moreover, healing tissue started growing in the perimeter of the initial lesion, with improved skin elasticity, less retraction, and less inflammation, finally resulting in healthy cicatricial tissues (Figure 3).

A summary of the clinicopathological features and the treatment regimen is presented in Table 1.

### 3.2. Case 2—T.T.

Patient T.T., female, 64 y.o., came to our Oncology Department in June 2021 for an ulcerated tumoral mass localized in the left breast that progressed over several months.

**Figure 4:**
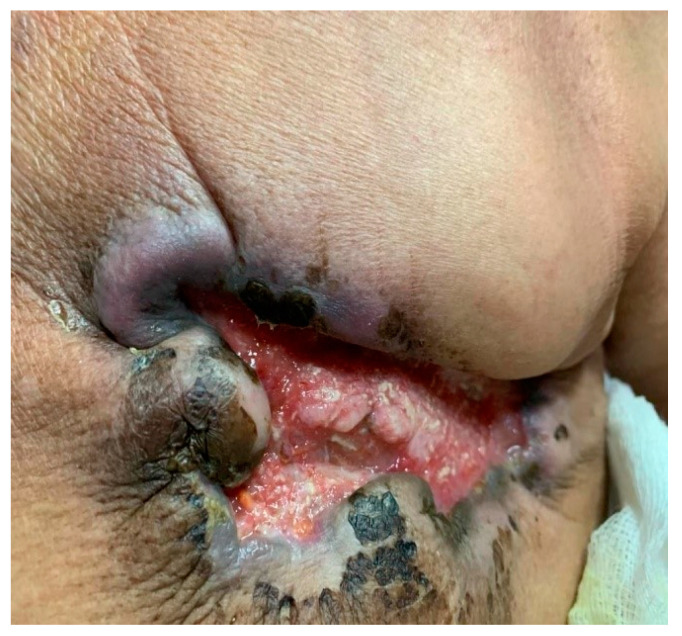
Clinical image of Case 2—T.T., June 2021. The figure illustrates approximately a 6 cm ulcerated tumor with irregular margins, skin changes, and involvement of the areola.

Clinical examination showed a 6 cm ulcerated tumor in the upper quadrant, with irregular margins and skin modifications; the lesion was located across all the left mammary glands, along with multiple lymph nodes (axillary and supraclavicular). The patient expressed moderate to intense pain in the left breast. She was a smoker, obese, with a body mass index (BMI) of 36, on anti-hypertensive treatment, and postmenopausal; additionally, there was no family history of cancer, no allergies, and no occupational exposure to carcinogens (Figure 4).

**Figure 5:**
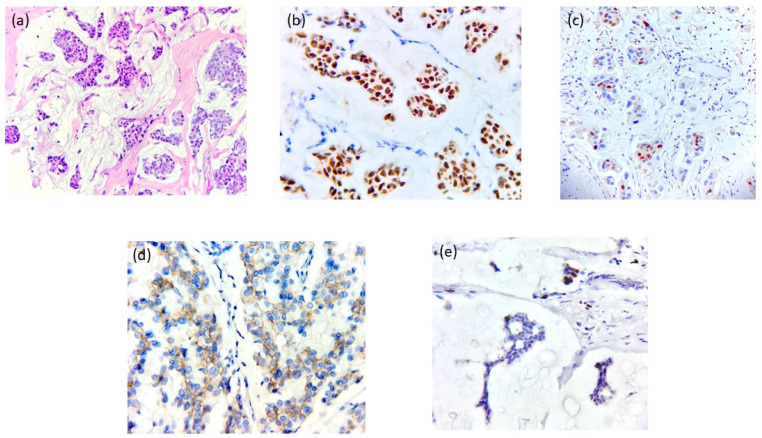
Immunohistochemistry of Case 2—T.T. (**a**) HE × 200: Mucinous breast carcinoma: nests of tumor cells floating in pools of extracellular mucin; (**b**) ER × 200: Estrogen receptor positive with strong nuclear expression in tumoral cells; (**c**) PR × 200: Progesterone receptor positive with variable nuclear expression in tumoral cells; (**d**) HER2 × 400: weak to moderate complete membrane staining observed in >10% of invasive tumor cells (score 2+/equivocal); (**e**) Ki67 × 200: Estimated Ki67 labeling index is 5%.

The biopsy exposed small ulcerated tissue fragments with polymorphic inflammatory infiltrate and neoformation vessels, presenting lesions with the appearance of invasive mucinous-type BC. Following IHC, the tumor expressed ER+, PR in 30% of the tumoral cells, and IHC = 2, with a Ki-67 proliferation index of 5% (Figure 5).

The whole-body CT scan showed a voluminous tumoral mass that completely invades the left mammary gland, with multiple enlarged left axillary lymph nodes, left infraclavicular and supraclavicular lymph nodes, and bilateral lymph nodes in the cardiophrenic angle; no visible secondary metastases of the parenchyma or bones have been observed. Osseous scintigraphy showed no malignant activity, only acute degenerative inflammatory changes of small joints of the hands and soles. Therefore, the diagnosis of BC, ER+, cT4dN2, with lymphatic dissemination (M1Lymp), stage IV, has been made.

Again, due to the extensive nature of the disease, the age of the patient, and its comorbidities, the case was debated by the MTD. As a result, we decided to use systemic therapy in addition to RT to preserve the QoL of the patient as much as possible while achieving an optimal therapeutic response. Therefore, the patient started with VMAT-RT on the left breast and left axillary lymph nodes, achieving a total dose of 50 Gy/25 fractions (2 Gy/fraction), followed by ET—letrozole, 2.5 mg/day p.o.—and CDK4/6 inhibitor palbociclib (125 mg/day p.o. for 21 days in a 28-day cycle, until the completion of RT). Additionally, the lytic bone lesions, skeletal-related events, and bone pain were managed by administering zoledronic acid (4 mg monthly via a 15-min infusion) and palliative VMAT-RT on the right pelvis target volume, achieving a total dose of 30 Gy/10 fractions (3 Gy/fraction). The patient also received dermatological care for local management, with mild radiation dermatitis treated using a moisturizer during and after radiotherapy. The CDK4/6 inhibitor was well tolerated, with occasional grade 1 neutropenia, according to the CTCAE severity scale, which did not necessitate any treatment interruptions.

**Figure 6:**
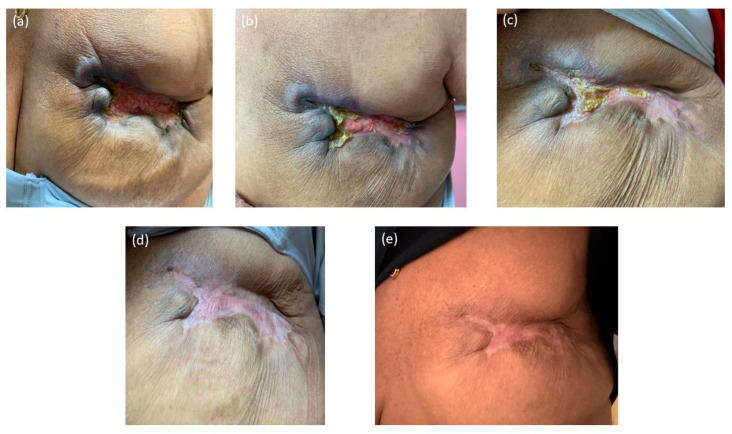
The clinical improvement of Case 2—T.T., after treatment completion, over a period of 12 months. (**a**) August 2021, one month after the treatment was initiated. (**b**) October 2021, the ulcerated tissue starts to reduce its dimensions, and the healing crusts begin to appear. (**c**) November 2021, a more intense cicatricial tissue is forming, while the initial lesion starts to diminish. (**d**) July 2022 and (**e**) December 2022, large post-radiotherapy changes and scarring, but no signs of secondary signs of breast cancer were identified.

Our expectations regarding the outcome were met. Following the treatment, the CT and positron emission computed tomography (PET-CT) display a significant reduction in the tumor mass in the left mammary gland. While the tumor extends in depth to the left pectoral mucosa, no cleavage plane has been observed in relation to it. Moreover, a reduction in the size of the left axillary, retropectoral, left supraclavicular, and right axillary tumor adenomegaly has been identified; no pulmonary, liver, or bone metastases were visible in the thoracic-abdominal-pelvic floor. During the next months, a clear improvement in the clinical aspect of the breast has been observed. The initial lesion reduced its diameter, while the process of neoformation of a healthy tissue began (Figure 6).

A summary of the clinicopathological features and the treatment regimen is presented in Table 2.

### 3.3. Review of Literature

The rationale for the MDT decision for these two cases presented is strengthened by personal experience and solid scientific data. Locally advanced or de novo stage IV breast cancer as the initial diagnosis is more and more common [4,5], and the treatment strategy for these patients is challenging. Traditionally, surgery of the primary tumor was conducted to avoid complications and for palliative symptoms (bleeding, ulcer, fungating lesion). Furthermore, radiotherapy can improve local control of advanced breast tumors. Taking into consideration that the overall survival of these patients is determined by the systemic and not the local disease, MDT discussion and collaboration are indispensable for long-term therapy strategy. To date, the removal of the primary tumor in patients with de novo stage IV breast cancer has not been associated with prolongation of survival, with the possible exception of the subset of patients with bone-only disease. However, it can be considered in selected patients, particularly to improve quality of life, always taking into account the patient’s preferences. The main purpose of this review of literature is to explore each specialist’s point of view during the MDT meeting.

#### 3.3.1. The Oncologist’s Point of View

In Romania, reimbursed standard treatment for HR-positive HER2-negative advanced breast cancer tumors is aligned with European Guidelines [13], which recommends a preferred first-line as CDK4/6 inhibitor plus ET. In light of this point, the authors will discuss options of standard treatments as the first choice. CDK4/6 inhibitors revolutionized the treatment of HR-positive HER2-negative advanced breast cancer. CDKs are crucial biological targets in the regulation of cell division, gene transcription, and biological processes [20,21,22,23,24]. The mechanism of action of CDK4/6 inhibitors relies on selective inhibition of the downstream CDK4/6-mediated phosphorylation of Retinoblastoma-associated proteins (pRb), leading to cell cycle arrest in the G0/G1 phase. During the early G1 phase of the highly controlled cell cycle, in response to mitogenic stimuli, several D cyclins bind and activate CDK4 and CDK6. Subsequently, the complex cyclin D-CDK4/6 selectively phosphorylates and inactivates members of pRb; therefore, Rb proteins limit the expression of many E2F target genes that stimulate the production of transcription factors that are involved in cell cycle progression, DNA replication, and mitotic progression [25,26,27,28].

Extensive research about both the mechanism of action and resistance to CDK4/6 inhibitors has been conducted in the last years since this vulnerability of cancer cells was discovered [29,30,31]. Currently, three CDK4/6 inhibitors—Palbociclib, Ribociclib, and Abemaciclib—are available and reimbursed by the Romanian National Insurance House (NHS); Palbociclib being the first on the Romanian market since 2019 [32]. Regarding Palbociclib, several studies exposed its efficacy. For example, phase III randomized clinical trials (RCTs) evaluated postmenopausal women with HR+, HER2-, advanced breast cancer (ABC). The Palbociclib + letrozole arm proved a statistically significant extended PFS compared to the placebo arm + letrozole (27.6 months versus 14.5 months), with a hazard ratio (HR) = 0.56, 95% confidence interval (CI) = 0.46–0.69, *p*-value < 0.000001 [4,5,33]. Additionally, the PALOMA-2 study presents similar results. In total, 179 out of 272 have been included in the Palbociclib + letrozole group. The results were impressive, with a disease-free interval (DFI) >12 months; median overall survival (OS) was 66.3 months (95% CI, 52.1–79.7), compared to the control group (47.4 months; 95% CI, 37.7–57.0) [34]. With respect to Riboclibi, the MONALEESA-2 study evaluated its results. The Ribociclib + letrozole arm had a PFS of 25.3 months vs. 16.0 months (placebo + letrozole arm). The treatment has been used as a 1st line therapy in ABC, with HR = 0.556 (95% CI, 0.43–0.72), statistically significant *p* < 0.00000329 [7]. Moreover, the latest results of the trial have been published in the ASCO 2022. Consequently, the OS was 63.9 months (inhibitor + letrozole) vs. 51.4 months (placebo + letrozole), HR = 0.76, 95% CI = 0.63–0.93, *p*-value = 0.004 [35]. Additionally, the MONALEESA-2 study exposed data that serve de novo metastatic disease or late recurrence group. In this case, the intervention arm achieved an OS of 68.2 months, compared to the control group (OS = 54.3 months) [36]. These results translated to a 25% relative reduction in the risk of death (HR = 0.75; 95% CI, 0.60–0.93; *p*-value = 0.005), consistent with the HR observed in the overall trial population. When adding ovarian suppression to the armamentarium for pre/perimenopausal women with HR+, HER2−, and ABC patients, the MONALEESA-7 study achieved outstanding results. The intervention group had a PFS of 23.8 months vs. 13.0 months in the placebo + endocrine therapy arm (HR = 0.55; 95% CI, 0.44–0.69; *p*-value < 0.0001) [8,37]; the updated reported median OS was 58.7 months with Ribociclib versus 48.0 months with placebo (HR = 0.76, 95% CI, 0.608–0.956) [38]. For Abemaciclib as a 1st line CDK4/6 inhibitor in HR+, HER−, and ABC, MONARCH-3 evaluated its results. Abemaciclib + letrozole achieved a PFS of 28.2 months compared to placebo + letrozole with 14.8 months (HR = 0.54; 95% CI, 0.418–0.698, *p*-value = 0.00002) [10,11]. Moreover, the latest and final OS data presented in a late-breaking session during the San Antonio Breast Cancer Symposium in December 2023 showed a median OS of more than 5.5 years in the intent-to-treat (ITT) population (66.8 vs. 53.7 months in the control arm), although statistical significance for the OS outcome was not reached (HR = 0.804; 95% CI, 0.637–1.015; *p*-value = 0.0664) [39,40].

Since both the Food and Drug Administration (FDA) and European Medicines Agency (EMA) approved CDK4/6 inhibitors in combination with ET, several systematic reviews and meta-analyses of RTCs were performed to aid in the clinical decisions based on the improvements in PFS, OS, and objective response rate (ORR) [41,42,43,44,45,46,47]. General conclusions on thousands of HR+, HER2−, and ABC patients included in one extensive analysis confirmed not only significantly longer PFS (HR = 0.55; 95% CI, 0.50–0.59; *p*-value < 0.00001) but also established an improvement in OS (HR = 0.79; 95% CI, 0.67–0.93; *p*-value = 0.004), irrespective of menopausal status, age, race, the presence of visceral metastasis. Above and beyond these encouraging data, CDK4/6 inhibitors manifested a significant advantage in the ORR (RR = 1.47; 95% CI, 1.30–1.67; *p*-value < 0.00001) and clinical benefit rate (CBR) (RR = 1.20; 95% CI, 1.12–1.30; *p* < 0.00001) [48]. The analysis conducted by Xu H. et al.l over 28 RTCs brought an even more interesting interpretation of the CDK4/6 inhibitors results, proving a superior efficacy in PFS but decreasing value when considering the treatment line [49].

Although databases selected for research are abundant in systematic reviews and meta-analyses of RTCs, real-life data are scarce. Schneeweiss A. et al. reported in 2020 the first experience of using CDK4/6 inhibitors in Germany after 2 years of drug availability, from the PRAEGNANT research network, since November 2016, three years earlier than in Romania [50]. Besides the increasing usage of CDK4/6 inhibitors plus ET rather than chemotherapy or ET monotherapy, the authors drew attention towards a more unfavorable prognosis profile of patients who were treated with this new therapy in real-life daily practice than in RTCs. Bofill S.J. et al. provided in 2022 their results from the Spanish sub-population of the phase 3b CompLEEment-1 trial [51]. The efficacy and safety of Ribociclib plus letrozole in HR+, HER2−, and ABC patients were evaluated, and the results were consistent with global data. A fairly small but relevant Romanian data extracted from a tertiary-level hospital real-world oncology practice revealed CDK4/6 inhibitors usage in both visceral and non-visceral ABC patients, with obviously different outcomes. Miron A.I. et al. recently published a five-year retrospective analysis of 107 HR+, HER2− ABC patients treated with CDK4/6 inhibitors plus ET in Romania, bringing to light an increased OS of these patients [52,53]. In conclusion, CDK4/6 inhibitors combined with ET in first-line HR+, HER2−, and ABC patients appear to be effective and relatively safe. Consequently, they are approved as standard of care by the ESMO guidelines [54] in harmony with systematic reviews and meta-analyses of RTCs and real-life setting data [55,56,57].

On the other hand, there is a substantial research interest in defining the intrinsic and acquired resistance mechanisms to CDK4/6 inhibitors plus ET in HR+, HER2−, and ABC patients [58,59]. The clinical efficacy of these novel therapies is undermined by different resistance pathways leading to disease progression [60,61]. The main resistance mechanisms are activation of certain pathways, such as Cyclin E-CDK2 or phosphoinositide 3-kinase (PI3K)/serine-threonine protein kinase (AKT)/mammalian target of rapamycin (mTOR) pathway activation, downregulation of pRP or phosphatase and tensin homolog (PTEN) and finally ET resistance [62,63,64]. Nevertheless, almost 10% of patients have primary resistance to CDK4/6 inhibitors [65]. One of the greatest challenges is distinguishing between mechanisms causing resistance to CDK4/6 inhibition and endocrine resistance. Many studies and clinical trials have found a connection between estrogen receptor 1 (ESR1) mutations and acquired resistance to endocrine therapy, ESR1 mutations being the most frequent and significant alterations resulting in resistance to ET [66,67,68]. However, no association was found between ESR1 and CDK4/6i resistance. Endocrine resistance and CDK4/6i sensitivity are associations worth discussing while deeply analyzing RTCs. PALOMA-3 [69], MONARCH-2 [70], and MONALEESA-3 [71] clinical trials confirmed that CDK4/6 inhibitors prolong PFS even after ET resistance, which validates that CDK4/6 inhibitors sustain effectiveness irrespective of the endocrine-resistant disease. Furthermore, endocrine-resistant tumors preserve sensitivity to CDK4/6 inhibitors, particularly when they are used in association with ET. In clinical practice, the understanding of CDK4/6 inhibitors’ mechanisms of action and resistance is crucial to better target predictive biomarkers of response, as intrinsic or acquired resistance could limit their value during the lifetime of ABC patients [72]. Numerous preclinical and translational research studies have been published in this line of interest to lighten the genomic and molecular landscape of resistance to these agents [73,74,75]. Asghar U.S. et al. concluded in a systematic review in 2022 that tumor profiling and next-generation sequencing (NGS) of liquid biopsies performed before starting CDK4/6 inhibition could be beneficial to clearly identify poorly responding patients [76]. Antonarelli G. et al.l provided updated research on clinically useful and relevant biomarkers of response or resistance to CDK4/6 inhibitors [77]. Regrettably, in Romania, access to these tests is restricted by unaffordable costs for most patients [58,78].

Clinical research and marketing approvals for the three available CDK4/6 inhibitors in Romania were timely and appropriate. To better comprehend and classify the right CDK4/6 inhibitor for the right patient, we performed a methodical analysis of the pharmacological differences between these three in-class medicines. To begin with, all three CDK4/6 inhibitors share a similar mechanism of action but with significant differences in terms of pharmacology [79]. All three are metabolized in the liver but with dissimilar half-lives: Palbociclib (29 h), Ribociclib (32 h), and Abemaciclib (18.3 h) [80,81,82]. As previously mentioned, all three drugs cause cell cycle arrest in the G1 phase, with Abemaciclib also affecting the G2 phase. Moreover, Abemaciclib has a broader target range, including CDKs 4 and 6, as well as CDKs 1, 2, 3, 5, 6, 9, 14, 16, 17, and 18; the later are less potent [83,84]. Regarding dosing schedule, these also vary: Palbociclib (125 mg once daily for 21 days, followed by a 7-day break), Ribociclib (600 mg once daily for 21 days), and Abemaciclib (150 mg twice daily continuously). Turning to side effects, these differ slightly. Myelosuppression is higher in Palbociclib and Ribociclib, while Abemaciclib has a milder effect. GI toxicity is more significant with Abemaciclib, whereas Palbociclib and Ribociclib present mild cases. Liver function test abnormalities are less frequent in Palbociclib but more present in Ribociclib and Abemaciclib. Furthermore, pneumonitis may be manifesting in all three therapies [85,86,87]. Ribociclib uniquely poses a risk of QT prolongation; 5% of patients in treatment groups experienced a QT interval above 480 ms. As a result, Ribociclib is recommended only for patients with QTcF below 450 ms, with EKG monitoring advised at baseline and early in treatment cycles. Palbociclib and Abemaciclib, by contrast, have not shown significant effects on QT prolongation. This profile highlights key considerations for clinicians when selecting a CDK4/6 inhibitor, especially regarding the management of hematologic, GI, hepatic, and cardiac risks [88,89].

As to QoL, adding CDK4/6 inhibitors to endocrine therapy generally preserves health-related quality of life (HR-QoL) in patients with breast cancer and even shows a trend toward pain improvement. Di Lauro et al. presented several findings in their work. Palbociclib combined with endocrine therapy provides comparable HR-QoL outcomes to endocrine therapy alone and appears superior to chemotherapy for HR-QoL. Ribociclib, studied in premenopausal women, shows similar HR-QoL scores to those observed in postmenopausal patients. However, while direct comparisons among the three CDK4/6 inhibitors are challenging due to study variations, these differences in side effects are crucial for tailoring treatment (especially in metastatic settings) to individual patient needs in clinical practice [90]. Moreover, specifically to Palbociclib, Kahan et al. evaluated the QoL using three types of questionnaires—European Organization for Research and Treatment of Cancer QLQ-C30, QLQ-BR23, and the EQ-5D-3L. They found that by the third treatment cycle, Palbociclib/ET patients showed an average QoL improvement of 2.9, compared to a −2.1 change for capecitabine patients (95% CI, 1.4–8.6; *p*-value = 0.007). Additionally, the median time to deterioration (TTD) in QoL was 8.3 months for Palbociclib/ET versus 5.3 months for capecitabine, with an adjusted hazard ratio of 0.70 (95% CI, 0.55–0.89; *p* = 0.003), indicating a longer period before decline for the Palbociclib/ET group; moreover, Palbociclib/ET also yielded superior results in other functional and symptom scales, including physical, cognitive, and social functioning, as well as fatigue, nausea/vomiting, and appetite loss [91]. In addition to these results, Harbeck et al. expose a novel eHealth technique to evaluate QoL. In their results, among the 412 patients (treated with Palbociclib/ET) evaluated for time to QoL deterioration, measured by a 10-point drop on the Functional Assessment of Cancer Therapy-General (FACT-G) score, the CANKADO-active group (the electronic-based patient-reported outcome) showed a significant advantage. Specifically, the cumulative incidence of QoL deterioration was lower in the CANKADO-active arm (HR = 0.698; 95% CI, 0.506–0.963); this was a pioneer eHealth trial demonstrating a significant benefit for mBC patients receiving oral tumor therapy when using an interactive autonomous patient empowerment application [92].

To conclude, the introduction of CDK4/6 inhibitors, specifically Palbociclib, has transformed treatment outcomes for HR-positive and HER2-negative advanced breast cancer. By targeting key mechanisms that regulate cell cycle progression and arresting tumor cells in the G0/G1 phase, Palbociclib enhances the efficacy of endocrine therapy and extends progression-free survival (PFS) for a significant period, as demonstrated in pivotal studies like PALOMA-2 and other phase III trials. Palbociclib, in combination with letrozole, offers a notable survival advantage with a median PFS nearly double that of endocrine therapy alone (27.6 vs. 14.5 months). Furthermore, Palbociclib consistently preserves QoL in metastatic settings, with patients reporting longer periods before symptom deterioration compared to other treatment modalities. Additionally, it is generally well-tolerated, with manageable side effects relative to alternative chemotherapy options. A summary of the results discussed above is presented in Table 3.

Given these significant benefits in PFS, overall survival, and patient-reported QoL metrics, Palbociclib remains a highly effective and recommended option for patients with HR+, HER2-advanced breast cancer, supported as a standard of care by both regulatory and clinical practice guidelines.

#### 3.3.2. The Surgeon’s Point of View

For centuries, mastectomy was used as a prophylactic measure in symptomatic infection to prevent its dissemination and, therefore, sepsis; later, mastectomy started to be utilized as a measure for clinically observed BC [93]. As the outcomes of bleeding control and infection improved, palliative mastectomy has been a common surgical option for managing advanced-stage breast cancer. However, this approach faces notable challenges, including the need to achieve clear surgical margins, often requiring a wide excision of skin. Additionally, securing adequate primary closure of the wound after achieving clear margins can be difficult, frequently necessitating complex reconstructive techniques [94,95]. Until recently, these technical difficulties and complications have been overcome by the necessity of the patient to survive. However, due to the recent developments in immunotherapy, palliative mastectomy is a subject of debate in the actual literature [96].

According to the American Society of Clinical Oncology (ASCO) 2024, palliative mastectomy is advised for patients with tumors that continue to bleed or ulcerate despite treatment, especially in settings with limited resources where RT is not available [97]. However, as previously stated, mastectomy can lead to physical challenges such as loss of sensation, changes in body image, and reduced sexual function; it also has a significant impact on mental health [98]. In terms of survival, there are data that suggest that primary excision of the tumoral mass in stage IV BC can improve this outcome. Khan et al. showed in their analysis of over 16,000 patients that surgery achieving free resection margins had a better prognosis when compared to no treatment or other palliative approaches (HR, 0.61; 95% CI, 0.58–0.65) [99]. Moreover, in locally advanced breast cancer (LABC), the standard of care begins with neoadjuvant chemotherapy (NACT). The rationale lies in the hope of reducing the tumoral volume and limiting its destructive effects, such as ulceration, bleeding, and infection. Therefore, a recent work conducted by Qin et al. focusing on the survival outcome in patients who underwent either mastectomy or conservative breast surgery (BCS) offered surprising results. In their study, the female patients with LABC who underwent a mastectomy after NACT had an improved 5-year overall survival (OS) when compared to BCS (OR, 2.6; 95% CI, 2.19–3.28; *p* < 0.00001) [100]. In addition, Lane et al. emphasize in their study that during the previous decade, the rates of surgery decreased because of systemic therapy. While the rates of surgery diminished, they showed that surgery—before (HR, 0.68; 95% CI, 0.62–0.73) or after (HR, 0.56; 95% CI, 0.52–0.61; *p* < 0.001) systemic therapy—achieved better OS than patients without surgery [101]. Similar results to the abovementioned studies are presented by Warschkow et al. In their propensity-adjusted study on over 16,000 patients, they found that surgery, as a treatment approach, decreased statistically significant from the beginning of the 21st century in mBC patients, while the overall mortality and cancer-specific mortality rates improved; in 1998, the HR for overall mortality was 0.72 (95% CI, 0.59–0.89), and in 2009, it improved to 0.42 (95% CI, 0.35–0.50), while for cancer-specific mortality, the HR was 0.72 (95% CI, 0.58–0.89) in 1998, and by 2009, it had further reduced to 0.40 (95% CI, 0.33–0.48) [102]. When stratified by immunohistochemistry, Douglas et al. identified 87,331 cases in their study. Surgical resection rates increased until 2009, reaching a peak of 37%, but declined to 18% by 2017. The most significant drop was in the HR+, HER2-negative subgroup, where surgery rates fell from 70% in 2007 to 15% in 2017. In 2004, systemic therapy alone was slightly more frequent than locoregional therapy (surgery and/or radiation) with or without systemic treatment (48% vs. 37%); by 2017, systemic therapy alone had become much more common (69% vs. 20%) [103].

On the other hand, early reports from prospective studies by Badwe et al. and Soran et al. did not show a survival benefit for de novo stage IV breast cancer patients who had surgical resection of the primary tumor [104,105]. Similarly, Sun et al. found in their study that patients with a good response to NACT had no significant difference in local recurrence (LR) or regional recurrence (RR) between a conservative approach and mastectomy (OR, 0.83; 95% CI, 0.60–1.15; *p*-value= 0.26; OR, 0.56; 95% CI, 0.33–0.93; *p*-value = 0.03). Moreover, breast-conserving surgery (BCS) was associated with a lower rate of distant recurrence (DR) (OR, 0.51; 95% CI, 0.42–0.63; *p*-value < 0.01), higher disease-free survival (DFS) (OR, 2.35; 95% CI, 1.84–3.01; *p*-value < 0.01), and better OS (OR, 2.12; 95% CI, 1.51–2.98; *p*-value < 0.01) compared with mastectomy [106]. Likewise, Nobrega et al. show in their study on over 900 patients that BCS achieved a higher pathological complete response (pR), OS, and lower distant metastases [107]. Notably, there is an age difference when deciding what type of treatment the patient will undergo. Elderly patients may not meet the criteria for surgery due to their cumulative comorbidities, while young people may not be restricted. For example, Hansen et al. present in their study on 210 patients that women with LABC aged 70 and older were less likely to receive neoadjuvant therapy as per guidelines, and only half of the patients in this age group underwent surgery with curative intent. However, there was no significant difference in 3-year OS or DFS between the younger and older age groups [108]. However, some findings were criticized for including a disproportionate number of patients with advanced metastatic disease, using insufficient systemic therapy regimens, and employing treatment sequences that did not align with modern practices. Hence, key results from Eastern Cooperative Oncology Group opened a phase III multicenter, prospective, randomized trial (ECOG 2108) indicated that while early locoregional therapy was associated with better local tumor control (16.3% vs. 39.8% in disease progression), it did not improve OS; median OS was 54.9 months for those receiving surgery compared to 53.1 months for those on systemic therapy alone, showing no significant difference. Additionally, there was no noticeable impact on patients’ QoL between the two groups, while more data are expected in 2025 [109].

Another significant aspect in patients with advanced BC is their QoL. While health is not just the absence of the disease but a more complex state of an individual, the treatment decision should encompass the patient’s needs in each sphere: physical, mental, and social well-being [110]. However, these needs are not met in every situation due to the disconnection of beliefs between the health practitioner and patient. For example, a significant portion of patients (≥64%) experiencing certain side effects from treatment do not discuss them with healthcare providers until they have a moderate or severe impact on their QOL. These side effects, including insomnia, diarrhea, back pain, and fatigue, can have a marked effect on daily functioning and overall well-being [111]. Hanson et al. compared satisfaction and well-being between those who had breast-conserving surgery with radiation therapy (RT) and those who underwent mastectomy with reconstruction but without RT. The analysis found no significant difference in breast satisfaction or physical well-being between the two groups. However, patients who had a mastectomy and reconstruction reported worse psychosocial (effect size, −8.61; 95% CI, −13.26 to −3.95; *p*-value < 0.001) and sexual well-being (effect size, −10.68; 95% CI, −16.60 to −4.76; *p*-value < 0.001) [112]. In addition, Diao et al. exposed in their study that patients who had mastectomy with reconstruction experienced worse psychosocial and sexual well-being compared to BCS+RT, though only the difference in sexual well-being was clinically significant. Moreover, older patients (≥65 y.o.) receiving BCS+RT and younger patients (<50 y.o.) with autologous reconstruction typically had better QOL scores [113].

On the other side, primary tumor resection did not extend overall survival for patients with advanced breast cancer when the tumor exceeded 5 cm in a study conducted by Shibasaki. However, in patients with a longer expected prognosis, surgery improved quality of life, offering some benefits in symptom management and comfort, even if it did not directly affect survival outcomes [114]. Moreover, Haussmann et al. evaluated the long-term QoL (mean follow-up of 17.7 years). The results showed that 85% of all patients rated their QoL as being positive. Specifically, 83% of those treated with BCS and 88% of those treated with mastectomy reported high levels of QoL, demonstrating a strong overall positive outcome for both groups [115]. The patient’s perception after breast reconstruction is worth mentioning. Fortunato et al. expose in their study that of the 328 patients who underwent breast reconstruction, 21% expressed regret or dissatisfaction with their decision [116]. Furthermore, Zeng et al. showed in their study that immediate breast reconstruction IBR in LABC was found to be significantly associated with an increased risk of both total surgical complications and major complications compared to mastectomy alone [117]. Therefore, major complications following immediate breast reconstruction (IBR) typically result in extended hospital stays, which can lead to delays in starting adjuvant therapies such as chemotherapy or radiation [118]. These delays can potentially affect the overall treatment timeline, causing both physical and psychological stress for patients.

A summary of the results regarding palliative surgery is presented in Table 4.

In summary, it has been shown that despite the historical reliance on surgery, studies now show that its benefits are mixed. Large-scale analysis revealed that surgery with clear margins improved survival in stage IV breast cancer, while other reports indicate that systemic therapies alone are becoming more common. Additionally, surgery in advanced cases still carries risks such as longer hospital stays and delayed adjuvant treatments. Interestingly, data comparing BCS to mastectomy show that BCS, especially following neoadjuvant therapy, is associated with lower distant recurrence rates, better disease-free survival, and improved overall survival. On the quality of life front, the picture is mixed. Many patients report satisfaction after both mastectomy and BCS, but psychosocial and sexual well-being often suffer more after mastectomy. This is further complicated by the fact that immediate breast reconstruction has been linked to a higher risk of complications, which can extend recovery and delay necessary therapies. While surgery remains important in some contexts, its role is being reevaluated in light of modern therapeutic advances, particularly for patients with advanced BC or mBC. The decision to operate must balance survival outcomes with QoL considerations, and therefore, a multidisciplinary team board is mandatory in this context.

#### 3.3.3. The Radiotherapist’s Point of View

As previously mentioned, women with LABC or inoperable local recurrence often endure a significantly reduced QoL due to the distressing symptoms that accompany advanced tumors. These tumors can cause chronic pain, bleeding, and ulceration, which can lead to severe physical discomfort. Additionally, tumor ulceration may result in malodorous discharge, which can exacerbate emotional distress and social isolation, further diminishing the patient’s overall well-being; pain from tumor invasion into surrounding tissues can be severe and difficult to manage, especially when conventional pain relief proves inadequate.

Concerning RT, it plays a crucial role in the multimodal management of BC, both in early-stage and locally advanced or metastatic cases [119,120]. However, in elderly patients with early disease, RT can be omitted. Additionally, in selected cases, RT of the whole breast is not mandatory, and accelerated partial breast irradiation (APBI) may be a better option [121]. Whole breast irradiation (WBI) is the standard method, typically administered post-surgery, to eliminate residual microscopic disease. APBI targets only the tumor bed, delivering higher doses over a shorter period, which can be advantageous for selecting patients with early-stage cancer. Chest wall irradiation is crucial for patients undergoing mastectomy, particularly those with locally advanced disease, to reduce recurrence risk. Lastly, irradiation of the lymphatic regions is often employed when there is a risk of nodal involvement, addressing potential metastases in lymph nodes. For example, the current RCT endorses the idea that patients with positive lymph nodes should be treated by radical mastectomy followed by hypofractionated RT targeting the chest wall, axillary apex, and supraclavicular area. Moreover, neoadjuvant systemic therapy should be followed by a mastectomy and postoperative radiation in patients with advanced stages (III–IV) or those with axillary [122,123,124].

Therefore, patients with mBC are increasingly benefiting from advanced radiation therapy techniques and new treatment paradigms [125]. Specifically, those with oligometastatic BC, whether synchronous or metachronous to the original diagnosis, may qualify for definitive treatment options, such as stereotactic body radiation therapy (SBRT). This approach focuses high doses of radiation on metastatic sites, aiming for maximal local control while minimizing damage to surrounding healthy tissue [126,127,128]. Moreover, recent studies have shown promising initial outcomes regarding local control and overall survival with SBRT in patients with oligometastatic disease. For instance, a study indicated that SBRT could effectively prolong PFS and provide symptomatic relief in select patients with oligometastatic BC [129]. Additionally, the National Comprehensive Cancer Network (NCCN) recognizes the role of stereotactic techniques as a viable option in the multidisciplinary management of breast cancer, particularly for patients with limited metastatic spread [130]. Yee et al. presented in their study that 54% of patients with ulceration and bleeding reported improvements following radiotherapy. Although 60% of patients experienced moist desquamation, there were no instances of grade 4 or 5 radiation dermatitis; the median locoregional progression-free survival was recorded at 12 months post-treatment, with no significant difference in PFS and OS between patients with and without distant metastases [131]. Additionally, Hoeltgen et al. present in their study that among their patients, 95% experienced symptom improvement post-RT, and 28.6% achieved a reduction in analgesic requirements according to the World Health Organization (WHO) scale; regarding safety, RT was well-tolerated, with side effects primarily classified as low-grade, with skin erythema and fatigue being the most frequent [132]. Moreover, Sousa et al. performed an analysis involving 76 patients with LABC. The study revealed that 18% achieved a complete response after neoadjuvant systemic radiotherapy (NART); the OS rate at five years was reported at 54%, with PFS at 61% and a pathological response (pR) [133]. On the other hand, Brackstone et al. report good results with chemotherapy concurrent with NART but with limited statistical significance. Of 32 patients, matched to 81 control patients, chemoradiation led to a significant increase in pCR rates (14% vs. 22%, *p*-value < 0.001). However, no significant differences were found in DFS (69% vs. 81%, *p*-value = 0.186) or OS (74% vs. 89%, *p*-value = 0.162) at three years; toxicity was also notable, with 25% of patients experiencing grade 3 pneumonitis and dermatitis, and one treatment-related death [134]. Furthermore, the reverse sequence of treatment, starting with NCHT followed by preoperative RT, mastectomy, and then IBR, is an emerging strategy for selecting patients with locally advanced breast cancer. In this regard, Maire et al. exposed in their study that the reverse sequence showed no significant differences in terms of OS and relapse-free survival (RFS); the five-year OS was 88.4% for the reverse sequence versus 81.5% for the standard approach (*p*-value = 0.4412), while the five-year RFS was 78.3% for reverse sequence and 70.1% for standard therapy (*p*-value = 0.3003) [135].

In terms of adjuvant RT, several aspects are of great interest. While neoadjuvant systemic therapy is increasingly used in BC, HER2-positive and triple-negative BC patients are showing higher rates of pCR. Consequently, managing the axilla after NST remains a crucial clinical question. For patients presenting with clinical N2/N3 disease, adjuvant RT with regional nodal irradiation (RNI) remains the standard approach, regardless of the response to treatment. Similarly, patients with clinical T4 BC are also candidates for adjuvant radiation following neoadjuvant systemic therapy. These treatments ensure adequate locoregional control and help reduce the risk of recurrence in high-risk populations [136]. Monten et al. investigated the safety and feasibility of external beam radiotherapy (EBRT) in patients over 65 y.o. The total dose ranged between 28.5 Gy and 34.5 Gy, depending on the target area (breast, thoracic wall, lymph nodes, or tumor bed). The primary concern was clinically significant dermatitis (grade ≥2), observed in 11.6% of cases. Dermatitis primarily occurred in patients receiving breast irradiation with a boost (17.5% grade 2–3) compared to no-boost patients, who had no recorded cases of grade ≥2 dermatitis; the study showed manageable toxicities even at higher doses [137]. Moreover, Lu et al. showed that late-course concurrent chemoradiotherapy (CCRT) significantly improved 3-year local-regional recurrence-free survival (LRFS) compared to short-course SCRT (92.3% vs. 81.8%, *p* = 0.046), although no significant differences were observed in 3-year DFS or OS. Notably, in the pT3-4 pN1-3 cM0 subgroup, both local recurrence-free survival and DFS were significantly better in the CCRT group (*p*-value = 0.036 and *p*-value = 0.049, respectively); no significant differences in adverse reactions between the two treatment groups, indicating that CCRT may offer enhanced local control without additional toxicity [138].

As previously mentioned, the surgical management of elderly patients with BC should not be compromised solely based on age because BCS may offer better QOL, according to several studies, compared to more radical approaches such as mastectomy. Therefore, avoiding extensive axillary surgery in favor of sentinel lymph node dissection further improves short-term QOL in elderly patients. Although conventional axillary surgery does not have a long-term QOL impact, advancements in RT have greatly enhanced tolerability, reducing late-onset tissue damage while maintaining effective cancer control; this paradigm-shifting makes treatment in elderly patients safer and more manageable using RT [139]. Gao et al. aimed to explore the evolution of health-related quality of life (HRQoL) in BC patients over a decade following radiotherapy RT. They found a slight decline in global health status during RT, followed by an improvement 6 weeks post-RT, before stabilizing near baseline at the 10-year mark. Most functional aspects either declined or remained unchanged after 10 years, except for role functioning, which improved. Dyspnea and diarrhea, however, worsened during this period. However, long-term global health status between survivors and unaffected women showed no significant differences, while specific issues like emotional distress, sleep problems, and fatigue were more pronounced in survivors, especially those under 65 [140]. Moreover, from the development of CDK4/6 inhibitors, studies are trying to evaluate their toxicity and QoL concurrent with RT. A work conducted by Kubeczko et al. showed a pooled prevalence of grade 3 toxicity at 30.6%, severe hematologic toxicity at 29.4%, and severe non-hematologic toxicity at 2.8% when combining targeted therapy with RT. Therefore, given that most radiotherapy RT treatments in their cases were prescribed with palliative intent, it is crucial to approach the use of higher radiation doses in combination with other therapies, such as CDK4/6 inhibitors, cautiously [141,142]. The refusal rate of the RT is worth mentioning in the actual context. Liu et al. showed an ascending trend of denying the RT. While their results showed that RT statistically significantly improved the OS, the refusal rate rose by 1% during the 2010–2015 period; several factors have been identified, such as age, net income, conjugal status, race, grade of the tumor and staging, molecular subtype and receiving chemotherapy or not [143].

Thus far, patients with LABC often suffer from painful symptoms such as chronic pain, bleeding, and ulceration, which greatly diminish QoL [144]. For early breast cancer, WBI is standard, but alternatives like APBI may be preferable for select cases. For patients with locally advanced disease, chest wall, and lymphatic irradiation are key to reducing recurrence risk, especially following mastectomy. New strategies, such as SBRT, offer promising outcomes for those with oligometastatic disease by targeting specific metastatic sites; recent studies also suggest that SBRT improves both PFS and OS in such patients. Hypofractionated treatments and intensity-modulated RT have shown improved tolerability and reduced side effects. For older patients, BCS coupled with sentinel lymph node dissection, rather than full axillary surgery, enhances QoL without sacrificing outcomes. Moreover, evidence also supports the use of neoadjuvant chemotherapy followed by RT, mastectomy, and reconstruction as a viable treatment sequence for some patients, with similar survival outcomes to traditional approaches. In addition, CCRT has been associated with better local control than sequential therapy, particularly in patients with high-risk breast cancer. However, managing toxicities remains a challenge, especially with combination therapies like CDK4/6 inhibitors. Caution is necessary when using high-dose RT in palliative settings, and efforts should be made to balance efficacy with the potential for severe side effects. Finally, while RT refusal rates have risen slightly in recent years, studies continue to demonstrate its critical role in improving OS.

## 4. Discussion and Conclusions

This study underscores the vital role of a multidisciplinary approach in managing ABC, emphasizing that treatment decisions benefit substantially from the input of an integrated team. In particular, a comprehensive plan combining systemic therapies—like CDK4/6 inhibitors and endocrine therapy—with surgical, radiological, and palliative care approaches enables personalized, evidence-based treatment pathways.

The cases reviewed demonstrate the importance of coordinating treatment for local control and symptom management, which directly enhances patients’ QoL. Radiotherapy plays an essential role, particularly in controlling symptomatic tumors and preventing further progression, while the incorporation of CDK4/6 inhibitors has improved PFS, delaying the need for more aggressive interventions. The balance between these interventions—guided by the combined expertise of oncologists, radiologists, surgeons, and support care specialists—ensures that treatment aligns with each patient’s unique clinical profile and personal preferences. This strategy not only addresses the immediate symptoms but also adapts to potential resistance mechanisms, thereby improving long-term outcomes and supporting continuous QoL monitoring. This multidisciplinary paradigm is recommended as a standard of care, supporting the latest guidelines and highlighting the potential for optimized outcomes when diverse specialties converge to tailor treatment for each patient.

In conclusion, this review acknowledged the benefits and limitations of both systemic treatment and local interventions, such as surgery and radiotherapy in ABC, and revealed the difficulty of making decisions during MDT meetings. Nevertheless, future research, including challenging patients as described above, is fundamental for more beneficial overall survival therapies and improved quality of life of ABC patients. Overwhelming CDK4/6 inhibitors randomized clinical trial results should be supported by real-life solid observational studies, where more frail patients would have the chance to be included. Forthcoming studies to explore the best-individualized lane would be relevant for all physicians included in managing ABC patients.

## Figures and Tables

**Table 1:** Clinicopathological features and treatment regimen of patient G.S.

Clinical Features		Pathological Features	Treatment Regimen
Tumor clinical characteristics and invasion	Other clinical characteristics	Invasive ductal carcinoma NST	VMAT—RT on left breast in a total dose of 30 Gy/10 fractions VMAT—RT on vertebral column bone metastasis in a total dose of 30 Gy/10 fractions
Central quadrant—left breast	BMI = 35	Poorly differentiated (G3)	Aromatase inhibitor Letrozole, 2.5 mg/day p.o. during RT
10 cm ulcerated, bleeding tumor	Postmenopausal	Estrogen receptor-positive	CDK4/6 inhibitor Palbociclib, 125 mg/day p.o. for 21 days in a 28-day cycle until RT completion
Multiple axillary lymph nodes	Anti-hypertensive treatment	Progesterone receptor-positive	Bisphosphonates Zoledronic acid, 15-min bolus, 4 mg/month
Moderate pain in the left breast + non-specific bone pain	History of hysterectomy and bilateral oophorectomy	HER2-negative	
cT4cN3c, pulmonary and osseous metastases		IHC score = 0	
Stage IV		Ki-67 proliferation index: 70%	

Abbreviations: BMI (Body Mass Index), NST (no special type), HER2 (Human Epidermal Growth Factor Receptor), IHC: (Immunohistochemistry), RT (Radiotherapy), CDK4/6 (Cyclin-Dependent Kinase 4/6).

**Table 2:** Clinicopathological features and treatment regimen of patient T.T.

Clinical Features		Pathological Features	Treatment Regimen
Tumor clinical characteristics and invasion	Other clinical characteristics	Invasive mucinous-type breast carcinoma	VMAT—RT on left breast in a total dose of 50 Gy/25 fractions VMAT—RT on right pelvis bone metastasis in a total dose of 30 Gy/10 fractions
Upper quadrant—left breast	Smoker	Estrogen receptor-positive	Aromatase inhibitor Letrozole, 2.5 mg/day p.o. during RT
6 cm ulcerated tumor	BMI: 36	Progesterone receptor (30% of tumoral cells)	CDK4/6 inhibitor Palbociclib, 125 mg/day p.o. for 21 days in a 28-day cycle until RT completion
Multiple lymph nodes (axillary and supraclavicular)	Postmenopausal	HER2-weak to moderate	Bisphosphonates Zoledronic acid, 15-min bolus, 4 mg/month
Moderate to intense pain in the left breast	On anti-hypertensive treatment	IHC score 2+/equivocal	
cT4dN2, with lymphatic dissemination		Ki-67 proliferation index: 5%	
Stage IV			

Abbreviations: BMI (Body Mass Index), HER2 (Human Epidermal Growth Factor Receptor), IHC: (Immunohistochemistry), RT (Radiotherapy), CDK4/6 (Cyclin-Dependent Kinase 4/6).

**Table 3:** CDK4/6 inhibitors in advanced breast cancer treatment.

Study	Treatment	Outcomes Measured	Results	
Finn et al. [4,5,33]	Palbociclib	PFS	Palbocilib + letrozole arm	Placebo + letrozole arm
			27.6 months	14.5 months
			HR = 0.56; 95% CI = 0.46–0.69; *p*-value < 0.000001	
PALOMA-2 [34]	Palbociclib	OS	Palbocilib + letrozole arm	Placebo + letrozole arm
			66.3 months (95% CI, 52.1–79.7)	47.4 months (95% CI, 37.7–57.0)
MONALEESA-2 [7,35]	Ribociclib	PFS	Ribociclib + letrozole arm	Placebo + letrozole arm
			25.3 months	16.0 months
			HR = 0.556; 95% CI, 0.43–0.72; *p*-value < 0.00000329	
		OS	63.9 months	51.4 months
			HR = 0.76; 95% CI = 0.63–0.93; *p*-value = 0.004	
MONALEESA-7 [8,37,38]	Ribociclib	PFS	Ribociclib + endocrine therapy	Placebo + endocrine therapy
			23.8 months	13.0 months
			HR = 0.55; 95% CI, 0.44–0.69; *p*-value < 0.0001	
		OS	58.7 months	48.0 months
			HR = 0.76, 95% CI, 0.608–0.956	
MONARCH-3 [10,11,39,40]	Abemaciclib	PFS	Abemaciclib + letrozole arm	Placebo + letrozole arm
			28.2 months	14.8 months
			HR = 0.54; 95% CI, 0.418–0.698; *p*-value = 0.00002	
		OS	66.8 months	53.7 months
			HR = 0.804; 95% CI, 0.637–1.015; *p*-value = 0.0664	
Li et al. [48]	CDK4/6 inhibitors + ET compared to ET alone	ORR	RR = 1.47; 95% CI, 1.30–1.67; *p*-value < 0.00001	
		CBR	RR = 1.20; 95% CI, 1.12–1.30; *p* < 0.00001	

Abbreviations: PFS (Progression-Free Survival), HR (Hazard Ratio), CI (Confidence Interval), OS (Overall Survival), ORR (Objective Response Rate), RR (Relative Risk), CBR (Clinical Benefit Rate).

**Table 4:** Summary of palliative surgery in terms of therapeutic approach and outcomes.

Study	Therapeutic Approach	Outcomes	Results		
			Experimental Arm	Conservative Arm	
Khan et al. [99]	Surgery	OS	Free-resection margins	No treatment/palliative	
			HR, 0.61; 95% CI, 0.58–0.65		
Qin et al. [100]	Surgery	5-year OS	Mastectomy after NACT	BCS	
			OR = 2.6; 95% CI, 2.19–3.28; *p*-value < 0.00001		
Lane et al. [101]	Surgery—before or after systemic therapy	OS	Before systemic therapy	Without surgery	
			HR, 0.68; 95% CI, 0.62–0.73		
			After systemic therapy	Without surgery	
			HR, 0.56; 95% CI, 0.52–0.61; *p*-value < 0.001		
Sun et al. [106]	Good response to NACT versus conservative approach/ mastectomy	LR	NACT	Conservative approach	Mastectomy
			OR, 0.83; 95% CI, 0.60–1.15; *p*-value= 0.26		
		RR	OR, 0.56; 95% CI, 0.33–0.93; *p*-value = 0.03		
		DR		OR, 0.51; 95% CI, 0.42–0.63; *p*-value < 0.01	
		DFS		OR, 2.35; 95% CI, 1.84–3.01; *p*-value < 0.01	
		OS		OR, 2.12; 95% CI, 1.51–2.98; *p*-value < 0.01	

Abbreviations: BCS (breast conservative surgery), NACT (neoadjuvant chemotherapy), OS (overall survival), HR (hazard ratio), CI (confidence interval), OR (odds ratio), DFS (disease-free survival), LR (local recurrence), RR (regional recurrence), DR (distant recurrence).

## Data Availability

Data are available on request due to ethical restrictions. The data presented in this study are available upon request to the corresponding author. The data are not publicly available due to the policy of the clinical hospital. Dr. Ion Cantacuzino has the approval of the Ethics Committee for each new research study.
